# A combination of metabolite profiling and network pharmacology to explore the potential pharmacological changes of secoisolariciresinol-diglycoside†

**DOI:** 10.1039/d0ra06382g

**Published:** 2020-09-21

**Authors:** Fengxiang Zhang, ShuangShuang Cui, Ziting Li, Yulinlan Yuan, Chang Li, Ruiman Li

**Affiliations:** Department of Gynaecology and Obstetrics, The First Affiliated Hospital of Jinan University Guangzhou 510632 China hqyylrm@126.com +86-20-38688603 +86-20-38688603; Institute of Traditional Chinese Medicine and Natural Products, College of Pharmacy, Jinan University Guangzhou 510632 China; Department of Medicinal Chemistry and Natural Medicine Chemistry, College of Pharmacy, Harbin Medical University Harbin 150081 China lichang661@126.com +86 451-86685745 +86 451-86685745

## Abstract

The prototypes and metabolites formed from the use of traditional Chinese medicines (TCM) are typically the cause of both side side-effects and therapeutic results. Therefore, the characterization of *in vivo* substances and the determination of functional changes are of great importance for clinical applications. Secoisolariciresinol-diglycoside (SDG), one major compound in flaxseeds, was used as a potential drug to treat tumors in the clinic; however, the metabolism information and functional changes of SDG *in vivo* were limited, which limited its application. In this study, an integrated strategy based on metabolite profiling and network pharmacology was applied to explore the metabolism feature and functional changes of SDG. As a result, a total of 28 metabolites were found in rats, including 14 in plasma, 22 in urine, 20 in feces, 7 in the heart, 14 in the liver, 8 in the spleen, 10 in the lungs, 14 in the kidneys, and 4 in the brain. Among them, M8, M13 and M26 were the main metabolites of SDG in rats and 24 were characterized for the first time. The metabolic reactions contained phase I reactions of demethylation, dehydroxylation, deglycosylation, arabinosylation and glycosylation, and phase II reactions of glucuronidation and sulfation were also observed. Notably, the arabinosylation and glycosylation were found in SDG for the first time. Meanwhile, 121 targets of SDG and its metabolites were found, PRKCB was the main target of SDG, and the metabolites of SDG mainly targeted HSP90A1, IL6, AKT1, MAPK3, MTOR, PIK3CA, SRC, ESR1, AR, PIK3CB, and PIK3CB. The difference of targets between SDG and its metabolites could result in its additional functional pathways of neurotrophin signaling pathway, PI3K-Akt signaling pathway, HIF-1 signaling pathway or indications of anti-prostate cancer. This work provided a new insight for exploring the mechanism and therapy indications of drugs.

## Abstract 2

The prototypes and metabolites formed from the use of traditional Chinese medicines (TCM) are typically the cause of both side side-effects and therapeutic results.

## Graphical abstract

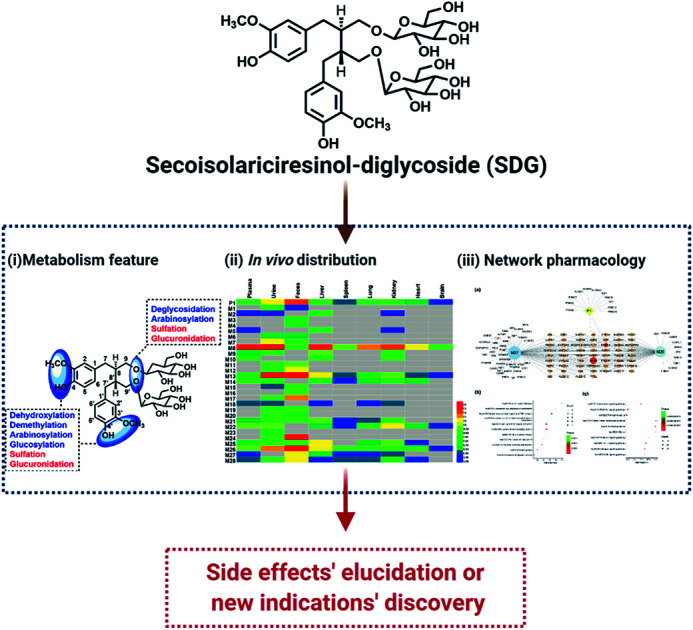

## 1. Introduction

Generally, components in traditional Chinese medicine (TCM) are absorbed into the circulatory system in the format of prototypes or metabolites after consumption, which contribute to therapy or side effects.^1^ Thus, characterizing the *in vivo* substances and the functional changes of TCM after administration is of great importance for its clinical applications. Meanwhile, good ADME (absorption, distribution, metabolism and excretion) and pharmacological properties are also essential and important to the clinical application of a natural product.^2^ Before that, the clear characterization of drug metabolism feature exerted great importance.

Secoisolariciresinol-diglycoside (SDG), a polyphenolic plant lignan, was characterized as one of the major compounds found in flaxseeds. It exerts serious activities of anti-depression,^3^ anti-tumor,^4^ anti-prostatic hyperplasia^5^ and analgesic effects,^6^*etc*. In previous work, the pharmacokinetic kinetics of SDG and its two metabolites (secoisolariciresinol and enterodiol) after oral administration were reported.^7^ Up to now, available data about metabolism of SDG *in vivo* was dramatically limited, although some researches indicated that it could be transformed into various metabolites by intestinal bacteria *in vitro*^8^ or some isolated metabolites in urine and feces.^9,10^ Moreover, the functional changes of SDG after administration was lost which limited its clinical application to avoid the side effects or develop new indications. Thus, it had urgent need to systematically profile the metabolites of SDG and its pharmacological changes *in vivo* in order to further pharmacological evaluation or clinical applications.

**Fig. 1:**
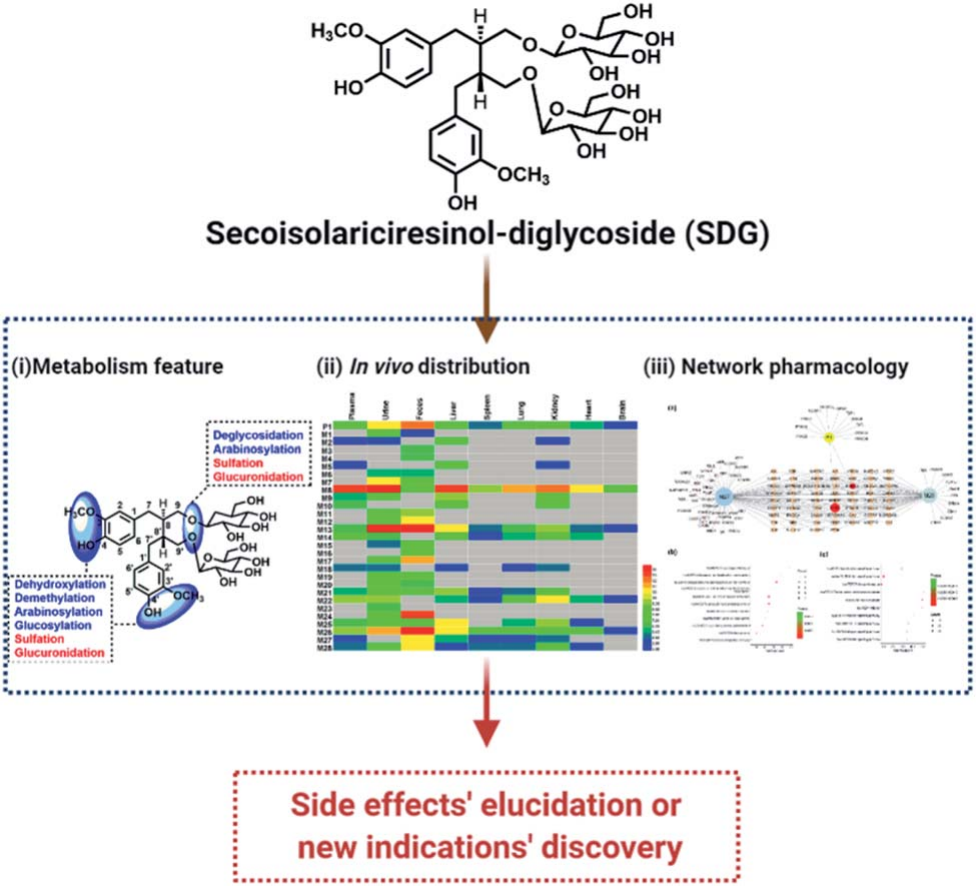
The flowchart of exploring pharmacological changes of SDG *in vivo*.

Nowadays, with the advantages of high sensitivity and resolution, high resolution mass spectrometry (HRMS) was widely used to profile and characterize the trace constituents of TCMs *in vitro* or their metabolites *in vivo*, such as Scutellariae Radix,^11^ Periplocae Cortex,^12^ Shuang-Huang-Lian oral liquid,^13,14^*etc*. Network pharmacology, with the advantages of systematically characterize the pharmacological index of complex system, was expansively applied in the field of TCM to reveal the pharmacological mechanism.^15^ Meanwhile, a combination of metabolites' profiling and network pharmacology was successfully applied in our previous work to characterize the functional changes of arctiin *in viv*o based on HRMS technology and network pharmacology.^1^ Thus, in this work, UHPLC-Q-TOF MS coupled network pharmacology was applied to characterize the metabolites of SDG and reveal their functional changes *in vivo* (Fig. 1).

## 2. Experimental

### 2.1 Materials

Secoisolariciresinol-diglycoside (SDG) and secoisolariciresinol, with purity more than 98% HPLC grade, were purchased from Chengdu Push Bio-Technology Co., Ltd. (Chengdu, China). HPLC grade water, methanol and ethanol and LC-MS grade acetonitrile and water were obtained from Fisher Scientific (Fair Lawn, New Jersey, USA). LC-MS grade formic acid was purchased from Sigma-Aldrich (St. Louis, USA).

### 2.2 Animals and drug administration

Specific pathogen Free male adult Sprague-Dawley rats (200–220 g) were obtained from Jinan Pengyue Experimental Animal Breeding Co. Ltd (Jinan, China). Rats were housed at ambient temperature of 20 ± 2 °C with 12 h light/dark cycles for two weeks, and in the meantime, a standard diet and water were free access. Rats were fasted in metabolic cages separately 12 hours before drug administration. The animal protocols were approved with the Guide for the Care and Use of Laboratory Animals of Jinan University, and procedures were in accordance with Guide for the Care and Use of Laboratory Animals (National Institutes of Health).

Twelve rats were classified into two groups, including control group (*n* = 3) and SDG group (*n* = 9). SDG groups were classified into three parts with three time-points, including 0.5 h, 1 h and 2 h group (*n* = 3). SDG group was administrated to SDG at gavage of 30 mg per kg per day; control group was administrated to equal volume of water. SDG was suspended in water as the final concentration of 7.5 mg mL^−1^.

### 2.3 Sample collection and pre-treatment

The rats (*n* = 9) were administrated with SDG (30 mg per kg per day) for three days. During this time, the feces and urine were collected, and they were stored at −80 °C before pre-treatment. On day 3, rats were sacrificed and the blood samples were collected after they were anesthetized by intraperitoneal injection of 10% aqueous chloral hydrate. The blood samples were collected from hepatic portal vein contained heparin sodium at 0.5, 1, 2 h after ingestion. Meanwhile, the organs (liver, heart, spleen, lung, kidney and brain) were also collected and washed by normal saline until there were not blood. Then, they were stored at −80 °C before pre-treatment.

#### 2.3.1 Blood

After centrifugation at 13 000 rpm for 10 min at 4 °C, 200 μL plasma of three different collected time was mixed and treated with acetonitrile at ratio of 1 : 4 to precipitated protein. The supernatant was obtained after centrifugation at 13 000 rpm for 10 min at 4 °C, and then, it was dried by nitrogen gas at room temperature. The residue was reconstituted in 300 μL 60% methanol–water (v/v).

#### 2.3.2 Urine

For pre-treatment, urine samples (10 mL) were thawing and centrifuging at 13 000 rpm for 10 min (4 °C), and 2 mL supernatant was loaded on a pre-activity HLB column (6 cm^3^, 200 mg, Waters Oasis, Ireland) directly. Then, it was orderly eluted by 6 mL of 5% methanol and 6 mL of methanol. The methanol eluate was collected and dried under nitrogen gas at room temperature. The residue was reconstituted in 300 μL 60% methanol–water (v/v).

#### 2.3.3 Feces samples

Feces were dried under the room temperature. Then, feces (10 g) were extracted by methanol and centrifuged at 13 000 rpm for 10 min (4 °C). Then, the supernatant was dried by nitrogen under room temperature and further reconstituted by water. The reconstituted samples were centrifuged by the same method, and 2 mL supernatants were loaded on a pre-activity HLB column (6 cm^3^, 200 mg, Waters Oasis, Ireland) directly, and then eluted by 6 mL of 5% methanol and 6 mL of methanol successively. The methanol eluate was collected and dried under nitrogen gas at room temperature. The residue was reconstituted in 600 μL 60% methanol–water (v/v).

#### 2.3.4 Heart, spleen, brain, lung and liver

The organs were weighted 2 g. Then, the collected tissues were homogenized by adding 2 mL normal saline, and treated with acetonitrile at ratio of 1 : 4 to precipitated protein. After centrifuging at 13 000 rpm for 10 min (4 °C), the supernatant was obtained and dried by nitrogen gas at room temperature. The residue was reconstituted in 300 μL 60% methanol–water (v/v).

The above samples were detected by UHPLC/Q-TOF MS, and three replicates were conducted. The collected MS information was analysed by Masslynx 4.1 (Waters, USA).

### 2.4 Instrumentation and conditions

A Waters Acquity™ ultra-performance LC system (Waters, Milford, USA) coupled an Acquity UHPLC BEH C18 Column (2.1 × 100 mm, 1.8 μm, Waters, Milford, USA, held at 35 °C) were used for chromatographic separation of samples. Two solvent mobile phases system, consisted of eluent A (0.1% formic acid in water, v/v) and eluent B (0.1% formic acid in acetonitrile, v/v), were used for separations, and delivered flow rate was set as 0.4 mL min^−1^ by using a liner gradient program. Detail information of this program was listed as follows: 0–2.0 min, 5% B; 2.0–5 min, 2–15% B; 5–10 min, 15–25% B; 10–11 min, 25–100% B; 11–12 min, 100% B; 12–12.5 min, 5% B; 12.5–13 min, 5% B.

A Waters Xevo™ G2-XS QTOF (Waters, Manchester, UK) was connected to the UPLC system *via* an ESI interface. The optimal conditions of analysis were set as follows: ESI^−^ mode, capillary voltage of 3 kV, sampling cone voltage was 35.0 V, extraction cone voltage was 4.0 V. The temperature was set at 100 °C, the desolvation gas temperature was 300 °C, and the desolvation gas flow was 800 L h^−1^. The full scan MS data were produced across the mass range of 50–1200 Da. Data were collected in centroid mode and the mass was corrected during acquisition using an external reference (Lock-Spray™) comprising a 200 pg mL^−1^ solution of leucine enkephalin *via* a lockspray interface, generating a reference ion at *m*/*z* 554.262 Da ([M − H]^−^) under the negative ion mode and *m*/*z* 556.277 Da under positive ion mode.

### 2.5 Target network analysis

The targets were retrieved and collected from online targets prediction platform Swiss Target Prediction (http://www.swisstargetprediction.ch)^16^ and similarity ensemble approach (SEA).^17^ Homon species was used for targets prediction. The protein–protein interactions (PPIs) were achieved by STRING database (version 11.0, https://string-db.org/),^18^ and protein interactions with a confidence score > 0.7 were selected in designed setting after eliminating duplicates. The chemical-target network and protein–protein interactions (PPIs) network were constructed by Cytoscape software (version 3.2.1).^19^ All proteins/genes were subjected to pathway enrichment analysis (KEGG analysis) using the DAVID Bioinformatics resources 6.7 database.^20^

## 3. Results and discussion

### 3.1 Fragmentation pathways of SDG

**Fig. 2:**
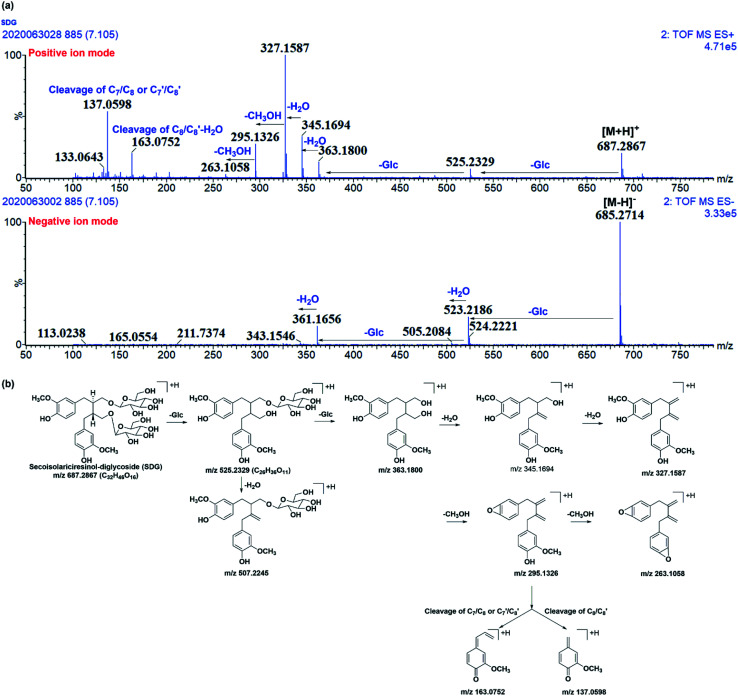
The mass fragmentation behaviours of SDG. (a) Mass chromatography of SDG under negative and positive ion mode; (b) proposed fragmentation pathways of SDG.

Before characterizing the metabolites of SDG in rats, its mass fragmentation behaviours were summarized. As shown in Fig. 2a, it was found that SDG presented obvious [M − H]^−^ at *m*/*z* 685.271 and [M + H]^+^ at *m*/*z* 687.287.

Apparently, it could lose glycosides to produced *m*/*z* 523.218 [M − H-C_6_H_10_O_5_]^−^ and 361.166 [M − H-2C_6_H_10_O_5_]^−^ under negative ions mode and fragments at *m*/*z* 525.233 [M + H-C_6_H_10_O_5_]^+^, 363.180[M + H-2C_6_H_10_O_5_]^+^ under positive ion mode, respectively. After losing two glycoses, the precursor could loss water to produce *m*/*z* 345.165 and 327.159 under positive ion mode. Moreover, the subsequently loss of CH_3_OH under positive ion mode resulted in fragment ions at *m*/*z* 295.133 and 263.106. Meanwhile, the cleavage of C7/C8 or C7′/C8′ and rearrangement could result in fragment ions at *m*/*z* 137.060; subsequently loss of water, the cleavage of C8/C8′, and rearrangement resulted in fragment ion at *m*/*z* 163.075 (Fig. 2a and b).

It was found that mass fragment ions of SDG were richer under positive ion mode than negative ion mode (Fig. 2a). Although the fragments under negative ion mode were few, but it was clear to identify the deprotonated ions. Then, the mass spectrometry information under both the positive and negative ion mode were used for screening and characterizing SDG-related metabolites.

### 3.2 Characterizing the metabolites of SDG in rats

**Fig. 3:**
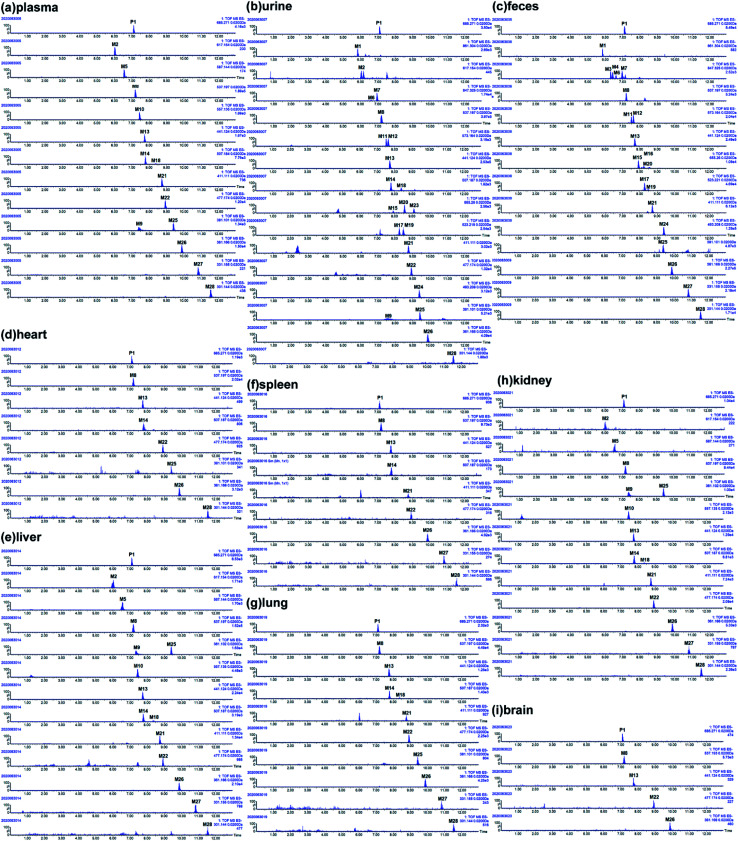
Extracted ion chromatography (EIC) of SDG and its metabolites in rats. (a) Plasma; (b) urine; (c) feces; (d) heart; (e) liver; (f) spleen; (g) lung; (h) kidney; (i) brain.

**Fig. 4:**
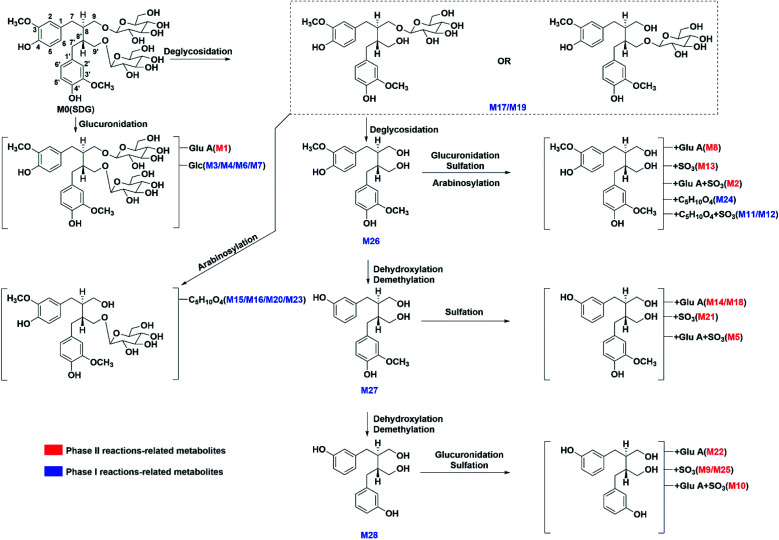
Proposed metabolism pathways of SDG in rats.

**Fig. 5:**
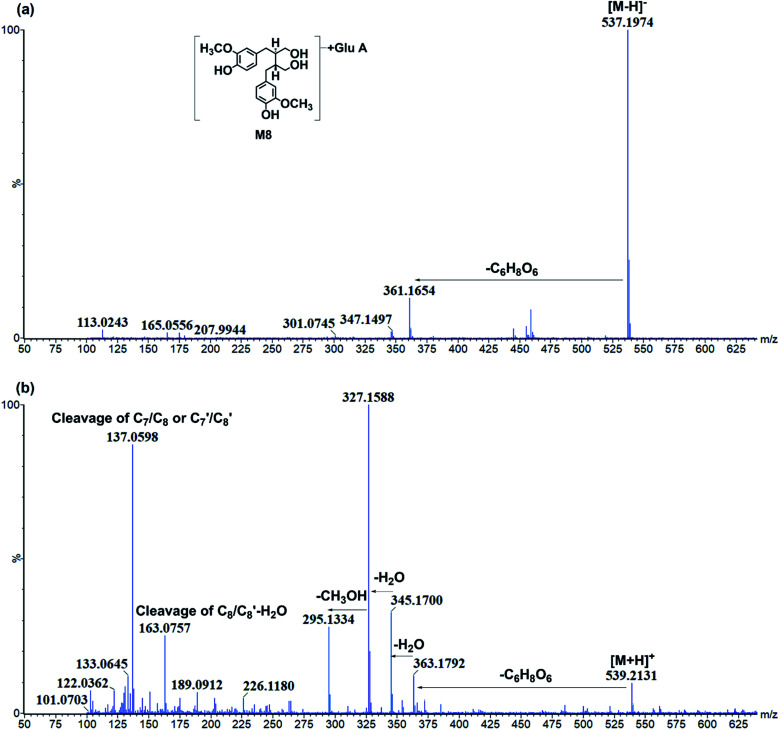
The representative chromatography of M8. (a) Negative ion mode; (b) positive ion mode.

The metabolites of SDG were screened out by using diagnostic ions extraction, prediction platform and the data in reported works.^7–10,21^ As a result, a total of 28 metabolites were found in rats, including 14 in plasma, 22 in urine, 20 in feces, 7 in heart, 14 in liver, 8 in spleen, 10 in lung, 14 in kidney, 4 in brain (Table 1, Fig. 3 and 4). The metabolic reactions contained phase I reactions of deglycosylation, glycosylation, demethylation, dehydroxylation, arabinosylation, and phase II reactions of glucuronidation, sulfation were also observed (Fig. 5). Among them, glycosylation and arabinosylation were reported for the first time.

**Table 1:** The metabolites identified or tentatively characterized in rats

No.	RT (min)	MS/error (ppm)	Formula	Fragment ions	Characterization	Origin
M0^a^	7.11	[M − H]^−^ 685.271(0.1) [M + H]^+^ 687.286(0.0)	C_32_H_46_O_16_	MS^−^: 523.219, 505.208, 361.166, 343.155, MS^+^:525.233, 363.180, 345.169, 327.159, 295.133, 163.075, 137.060	Secoisolariciresinol-diglycoside (SDG)	P, U, F, H, Li, S, Lu, N, B
M1	5.84	[M − H]^−^ 861.304(1.5) [M − H]^+^ 863.317(−1.3)	C_38_H_54_O_22_	MS^−^: 685.270, 361.165, MS^+^: 327.157	Secoisolariciresinol-diglycoside glucuronide	U, F
M2	6.00	[M − H]^−^ 617.154(0.8)	C_26_H_34_O_15_S	MS^−^: 537.191, 441.123, 361.165	Secoisolariciresinol- glucuronide sulfate	P, U, Li, N
M3	6.31	[M − H]^−^ 847.323(−0.7) [M + H]^+^ 849.341(1.8)	C_38_H_56_O_21_	MS^−^: 685.266, 523.2175, 361, MS^+^: 687.285, 525.234, 363.180, 345.170, 327.159, 295.133, 163.076, 137.060	Secoisolariciresinol-triglycoside	F
M4	6.42	[M − H]^−^ 847.323(−0.8) [M + H]^+^ 849.339(−0.8)	C_38_H_56_O_21_	MS^−^: 685.264, 523.221, MS^+^: 327.159, 295.133	Secoisolariciresinol-triglycoside	F
M5	6.57	[M − H]^−^ 587.145(1.7)	C_25_H_32_O_14_S	MS^−^: 507.189, 411.112, 331.152	Demethyl-dehydroxyl-secoisolariciresinol glucuronide sulfate	P, Li, N
M6	6.61	[M − H]^−^ 847.320(−4.8) [M + H]^+^ 849.341(2.6)	C_38_H_56_O_21_	MS^−^: 685.271, 523.219, 361.166, MS^+^: 327.160, 295.132, 163.075, 137.060	Secoisolariciresinol-triglycoside	U, F
M7	6.93	[M − H]^−^ 847.325(1.5) [M + H]^+^ 849.337(−2.9)	C_38_H_56_O_21_	MS^−^: 685.270, 667.262, 523.217, 361.165, MS^+^: 345.168, 327.160, 295.132, 163.074, 137.060	Secoisolariciresinol-triglycoside	U, F
M8	7.20	[M − H]^−^ 537.197(0.2) [M + H]^+^ 539.215(4.3)	C_26_H_34_O_12_	MS^−^: 361.165, MS^+^: 363.179, 345.170, 327.159, 295.134, 163.076, 137.060	Secoisolariciresinol glucuronide	P, U, F, H, Li, S, Lu, N, B
M9	7.34	[M − H]^−^ 381.102(2.6)	C_18_H_22_O_7_S	MS^−^: 301.145	Enterodiol-3-*O*-sulfate or enterodiol-3′-*O*-sulfate	P, U, Li, N
M10	7.43	[M − H]^−^ 557.135(2.9)	C_24_H_30_O_13_S	MS^−^: 381.102, 301.145	Enterodiol sulfate glucuronide	P, Li, N
M11	7.50	[M − H]^−^ 573.164(0.2)	C_25_H_34_O_13_S	MS^−^: 493.208, 361.165	Secoisolariciresinol arabinofuranoside sulfate	U, F
M12	7.62	[M − H]^−^ 573.168(−2.4)	C_25_H_34_O_13_S	MS^−^: 493.209, 361.164	Secoisolariciresinol arabinofuranoside sulfate	U, F
M13	7.73	[M − H]^−^ 441.124(1.1)	C_20_H_26_O_9_S	MS^−^: 361.166, 346.142	Secoisolariciresinol sulfate	P, U, F, H, Li, S, Lu, N, B
M14	7.78	[M − H]^−^ 507.187(0.6)	C_25_H_32_O_11_	MS^−^: 331.155	Demethyl-dehydroxyl-secoisolariciresinol glucuronide	P, U, H, Li, S, Lu, N
M15	7.91	[M − H]^−^ 655.261(0.5) [M + H]^+^ 657.270(−2.7)	C_31_H_44_O_15_	MS^−^: 523.218, 361.165, MS^+^: 327.159, 295.233, 137.058	Secoisolariciresinol-glycoside arabinofuranoside	U, F
M16	8.22	[M − H]^−^ 655.261(1.1)	C_31_H_44_O_15_	MS^−^: 523.220, 361.167	Secoisolariciresinol-glycoside arabinofuranoside	F
M17	8.26	[M − H]^−^ 523.218(−0.6) [M + H]^+^ 525.231(−4.8)	C_26_H_36_O_11_	MS^−^: 361.165, 346.141, MS^+^: 327.160, 295.133, 137.060	Secoisolariciresinol-glycoside	U, F
M18	8.37	[M − H]^−^ 507.188(3.5)	C_25_H_32_O_11_	MS^−^: 331.156	Demethyl-dehydroxyl-secoisolariciresinol glucuronide	P, U, Li, Lu, N
M19	8.46	[M − H]^−^ 523.221(6.3) [M + H]^+^ 525.233(−2.1)	C_26_H_36_O_11_	MS^−^: 361.165, MS^+^: 327.156, 137.060	Secoisolariciresinol-glycoside	U, F
M20	8.55	[M − H]^−^ 655.257(−5.3)	C_31_H_44_O_15_	MS^−^: 361.165	Secoisolariciresinol-glycoside arabinofuranoside	U, F
M21	8.75	[M − H]^−^ 411.111(−0.7)	C_19_H_24_O_8_S	MS^−^: 331.155, 165.0560	Demethyl-dehydroxyl-secoisolariciresinol sulfate	P, U, F, Li, S, Lu, N
M22	8.92	[M − H]^−^ 477.174(−3.8) [M + H]^+^ 479.189(−6.3)	C_24_H_30_O_10_	MS^−^: 301.1460, MS^+^: 107.050	Enterodiol glucuronide^9^	P, U, H, Li, S, Lu, N, B
M23	9.11	[M − H]^−^ 655.259(−2.6)	C_31_H_44_O_15_	MS^−^: 361.162	Secoisolariciresinol-glycoside arabinofuranoside	U
M24	9.38	[M − H]^−^ 493.208(1.8) [M + H]^+^ 495.224(1.6)	C_25_H_34_O_10_	MS^−^: 361.166, MS^+^: 363.178, 345.170, 327.160, 295.134, 163.076, 137.060	Secoisolariciresinol arabinofuranoside	U, F
M25	9.41	[M − H]^−^ 381.101(0.3)	C_18_H_22_O_7_S	MS^−^: 301.144, 271.132, 253.123	Enterodiol-9-*O*-sulfate or enterodiol-9′-*O*-sulfate	P, U, F, H, Li, Lu, N
M26^a^	9.88	[M − H]^−^ 361.166(3.0) [M + Na]^+^ 385.161(−4.1)	C_20_H_26_O_6_	MS^−^: 346.163, MS^+^: 327.195, 295.124, 163.076, 137.059	Secoisolariciresinol^8–10^	P, U, F, H, Li, S, Lu, N, B
M27	10.83	[M − H]^−^ 331.155(1.5)	C_19_H_24_O_5_	MS^−^: 165.055, 149.060	Demethyl-dehydroxyl-secoisolariciresinol^8,9^	P, F, Li, S, Lu, N
M28	11.53	[M − H]^−^ 301.144(0.0)	C_18_H_22_O_4_	MS^−^:271.132, 253.123	Enterodiol^8,10^	P, U, F, H, Li, S, Lu, N

a Identified by comparison with reference standards; RT-retention time, P-plasma, U-urine, F-feces, H-heart, Li-liver, S-spleen, Lu-lung, N-nephridium, B-brain.

#### 3.2.1 Deglycosylation metabolites of SDG

Three deglycosylation metabolites of SDG were detected in rats. M17/M19, detected in rat urine and feces, presented [M − H]^−^ at *m*/*z* 523.218 with the formula of C_26_H_35_O_11_. It had fragment ion at *m*/*z* 361.165 which was the diagnostic ion of precursor of SDG. As compared to SDG, M17/M19 lacked C_6_H_10_O_5_, and they were characterized as the products of SDG by loss of one glucosyl. M26 presented [M − H]^−^ at *m*/*z* 361.166 and [M + H]^+^ at *m*/*z* 363.161 which was the same as the precursor of SDG. With the help of characteristic ions under positive ion mode (*m*/*z* 327.195, 295.124, 163.076, 137.059) which was similar with the fragments of SDG, M26 was characterized as the metabolites of SDG by losses of two glucosyls.^9^ It was identified as secoisolariciresinol by comparison with reference standards.

#### 3.2.2 Demethylation and dehydroxylation metabolites of SDG

Two metabolites (M27/M28) were classified into demethylation and dehydroxylation-related group. M27 presented [M − H]^−^ at *m*/*z* 331.155 with the formula of C_19_H_23_O_5_. As compared to M26, it lacked CH_3_O. Then, M27 was characterized as dimethyl-dehydroxyl-secoisolariciresinol.^9^M28 presented [M − H]^−^ at *m*/*z* 301.144 with the formula of C_18_H_21_O_4_. As compared to M26, M28 lacked two CH_3_O and then, it was characterized as enterodiol.^9^

#### 3.2.3 Glycosylation or arabinosylation-related metabolites of SDG

##### Glycosylation-related

Four glycosylation-related metabolites (M3/M4/M6/M7) were found in rat urine and feces. They all presented [M − H]^−^ at *m*/*z* 847.323, with the formula of C_38_H_55_O_21_. The fragment ions at *m*/*z* 685.266, 523.221, 361.166 under negative ion mode were produced by subsequently losses of three glycosyl groups from precursor. Then, M3/M4/M6/M7 were characterized as secoisolariciresinol-triglycoside. The precursor, SDG, already had two glycoses and only two hydroxyls were suitably attached to glycosyl. However, there were four glycosylation-related metabolites. It might be achieved through the reactions of deglycosylation and glycosylation.

##### Arabinosylation-related

Five metabolites only with arabinose conjunctions were characterized in rat urine or feces, including M15/M16/M20/M23/M24. M15/M16/M20/M23 both presented same [M − H]^−^ at *m*/*z* 655.261, with the formula of C_31_H_43_O_15_. The fragment ions at *m*/*z* 523.218 and 361.165 were produced by losses of C_5_H_10_O_4_ (132 Da) and C_6_H_10_O_5_ (162 Da), indicating that these metabolites had arabinose and glycosyl conjunctions. Then, they were characterized as secoisolariciresinol-glycoside arabinofuranoside. M24 presented [M − H]^−^ at *m*/*z* 493.2083, with formula of C_25_H_33_O_10_. Meanwhile, the fragment ions at *m*/*z* 361.166, 165.056, 147.043 were produced by losses of C_6_H_10_O_5_ (162 Da), indicating that M24 had glycosyl conjunctions and the same parent structure as secoisolariciresinol. Thus, M24 was characterized as secoisolariciresinol arabinofuranoside.

#### 3.2.4 Phase II-related metabolites of SDG

##### Glucuronidation-related metabolites

The glucuronidation of SDG could result in metabolite M1 which had [M − H]^−^ at *m*/*z* 861.304 with the formula of C_38_H_53_O_22_. There were two sites of SDG for glucuronidation, including hydroxyl at C-4 and C-4′ position. The Clog *P* values of C-4 or C-4′ conjunctions were the same, and it was hard to distinguish under current chromatography conditions. Then, M1 was tentatively characterized as secoisolariciresinol-diglycoside glucuronide. Moreover, the glucuronidation of M26 resulted in metabolite M8. M8 had [M − H]^−^ at *m*/*z* 537.197 (C_26_H_33_O_12_) and fragments at *m*/*z* 361.165[M − H-C_6_H_8_O_6_]^−^ (Fig. 5). It had C_6_H_8_O_6_ (176 Da) more than M26, and then, it was characterized as secoisolariciresinol glucuronide. M14/M18 presented [M − H]^−^ at *m*/*z* 507.184 (C_25_H_31_O_11_) and fragment ions at *m*/*z* 331.155 [M − H-C_6_H_8_O_6_]^−^. Among them, *m*/*z* 331 was the deprotonated ion of M27, and they had the same fragmentation pathway. Then, M14/M18 were characterized as dimethyl-dehydroxyl-secoisolariciresinol glucuronide. M22 had [M − H]^−^ at *m*/*z* 477.174 (C_24_H_29_O_10_) and fragment ions at *m*/*z* 301.146, 253.122. It had similar fragmentation pathway as M28. The difference was that M22 had glucuronide conjunction more than M28. Then, M22 was characterized as enterodiol glucuronide.

##### Sulfation-related metabolites

M13 presented [M − H]^−^ at *m*/*z* 441.124 (C_20_H_25_O_9_S), and its fragment ions at *m*/*z* 361.166, 346.142, 165.056 under negative ion mode was similar to M26. Then, it was characterized as secoisolariciresinol sulfate. M21 had [M − H]^−^ at *m*/*z* 411.111 (C_19_H_23_O_8_S) and fragment ions at *m*/*z* 331.1545, 165.0559. Then, it was characterized as dimethyl-dehydroxyl-secoisolariciresinol sulfate. M9/M25 both had deprotonated ion at *m*/*z* 381.101 (C_18_H_21_O_7_S), and fragment ions at *m*/*z* 301.144, 271.132, 253.123. It had the same fragment ions as M28 which was characterized as enterodiol. The difference was that M9/M25 had sulfate more than M28. Then, they were characterized as the sulfation products of M28. However, the sulfate sites could not be unambiguously determined since there were four possible sites, including the hydroxyl of C-3, C-3′, C-9 and C-9′. With the help of Clog *P* values calculated in ChemDraw software (Cambridge, UK), M9 and M25 were tentatively characterized as enterodiol-3/3′-*O*-sulfate (Clog *P* = −0.42) and enterodiol-9/9′-*O*-sulfate (Clog *P* = −1.07).

##### Metabolites with glucuronide and sulfate conjunctions

M2 had [M − H]^−^ at *m*/*z* 617.155 (C_26_H_33_O_15_S) and fragment ions at *m*/*z* 537.191 [M − H-SO_3_]^−^, 441.123 [M − H-C_6_H_8_O_6_]^−^ and 361.165 [M − H-C_6_H_8_O_6_–SO_3_]^−^. It was characterized as secoisolariciresinol glucuronide sulfate. M5 had [M − H]^−^ at *m*/*z* 587.145 (C_25_H_31_O_14_S) and fragment ions at *m*/*z* 507.189 [M − H-SO_3_]^−^, 411.112 [M − H-C_6_H_8_O_6_]^−^ and 331.152 [M − H-C_6_H_8_O_6_–SO_3_]^−^. It was characterized as dimethyl-dehydroxyl-secoisolariciresinol glucuronide sulfate. M10 presented [M − H]^−^ at *m*/*z* 557.135 (C_24_H_29_O_13_S) and fragment ions at *m*/*z* 381.102 [M − H-C_6_H_8_O_6_]^−^, 301.145 [M − H-C_6_H_8_O_6_–SO_3_]^−^. Thus, it was characterized as enterodiol glucuronide sulfate.

##### Metabolites with arabinose and sulfate conjunctions

M11/M12 both had [M − H]^−^ at *m*/*z* 573.164 (C_25_H_33_O_13_S) and fragment ions at *m*/*z* 361.165 [M − H-C_5_H_8_O_4_–SO_3_]^−^. They were characterized as secoisolariciresinol arabinoside sulfate.

**Fig. 6:**
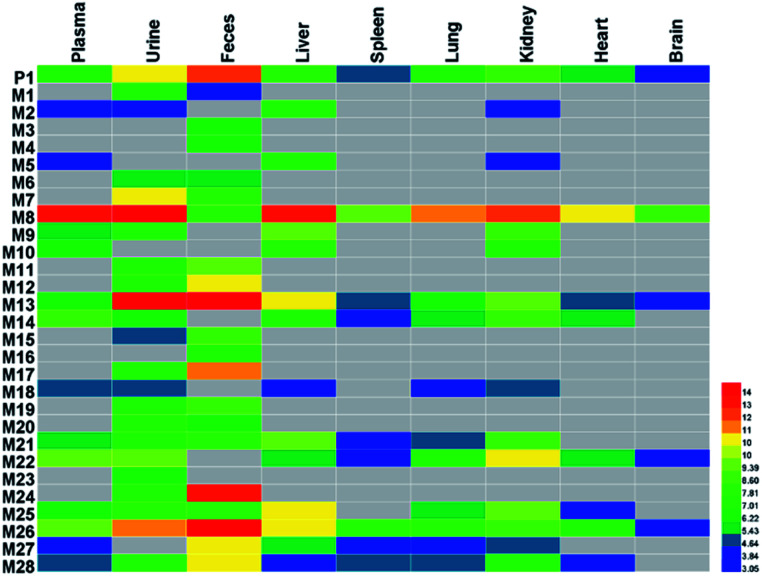
The heatmap of related distribution of SDG and its metabolites in rats. The color was correlated to the degrees of relative peak area in rats' biosamples, the deep color with red means high peak area and gray means that no metabolites were detected.

### 3.3 Metabolism feature of SDG

The SDG and its metabolites in rats' different tissue were also analysed (Fig. 6), and it was found that SDG could be detected in all collected samples of rats (plasma, urine, feces, liver, spleen, lung, kidney, heart, brain). The metabolites of SDG were mainly distributed in rats' plasma, urine, feces, liver and kidney which were the general metabolism or excretion places. For different metabolites, glycosylation-related metabolites were also presented in rats' plasma, urine, feces, liver and kidney; while, arabinosylation-related metabolites were only found in rat' urine and feces, indicating that the enzyme of arabinosylation might be in intestine. In rats' spleen, lung, heart and brain, only M26, M27 and their sulfate, glucuronide or glycoside conjunctions were found, indicating that these metabolites might be the functional basis of SDG treating the disease in these targets, such as tumor in lung.

**Fig. 7:**
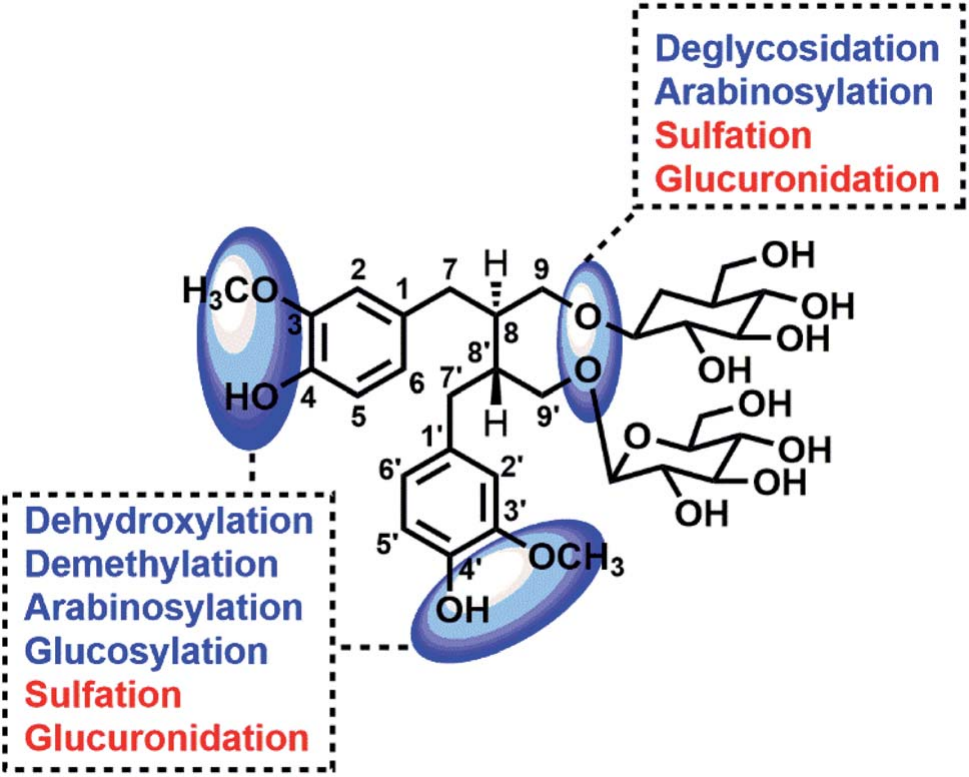
Potential sites for metabolic changes of lignans in flaxseed.

Through summarizing the metabolites of SDG, its metabolism feature was also characterized. Among them, M8, M13 and M26 were the main metabolites of SDG in rats. It was found that deglycosylation, one of general metabolic reactions for compounds with *O*-glycosides, was also found in SDG. Phase I reactions of glycosylation, arabinosylation, dehydroxylation, demethylation and phase II reactions of glucuronidation, sulfation were detected (Fig. 7). The metabolic reactions were generally occurred at C9/C9′, C3/C4 or C3′/C4′. As compared to the reported metabolic reactions of SDG,^9^ glycosylation and arabinosylation were reported for the first time.

### 3.4 Targets information of SDG and its metabolites

**Fig. 8:**
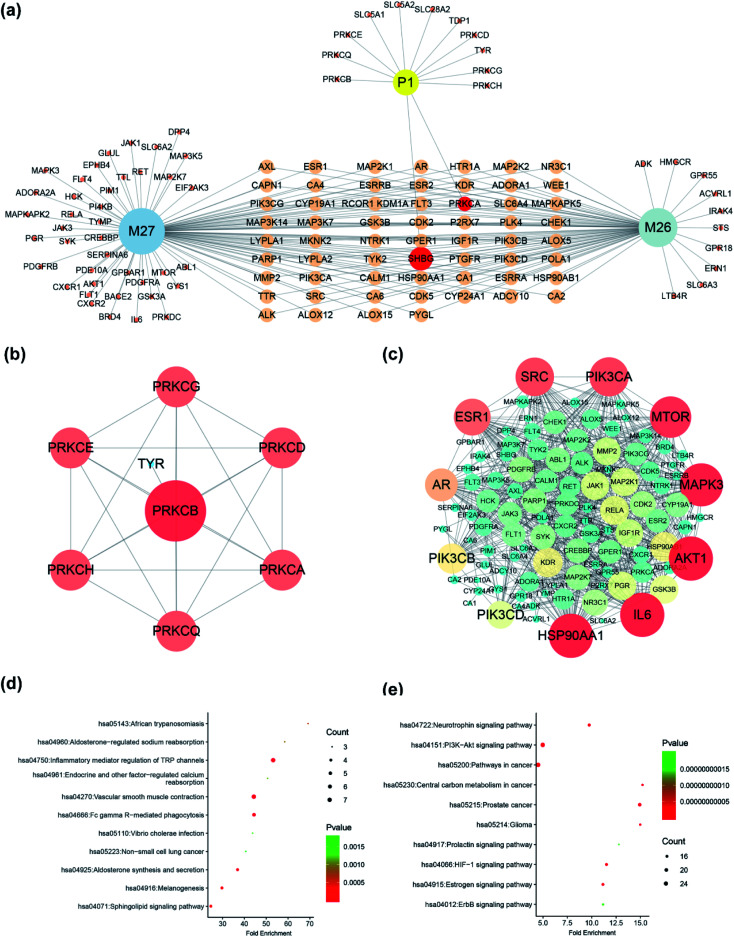
The network pharmacology of SDG and its metabolites. (a) Targets of SDG (P1) and its metabolites (M26 and M27); PPI of SDG (b) and metabolites (c); KEGG pathways of SDG (d) and its metabolites (e). The size and color were correlated to the degrees of targets in network: the big size and deep color with pink means high degree of this target in network.

Four compounds (P1, M26, M27, M28) were selected to construct pharmacological network since they had unambiguously identified structure. A total of 121 targets of three compounds (P1, M26, M27), with the probability more than 0.1, were obtained in Swiss Target Prediction and SEA; and then, compounds-targets network was constructed by using Cytoscape software. As shown in Fig. 8a, the network consisted of 124 nodes and 182 interactions. Two targets were presented both in SDG and its metabolites, including PRKCA and SHBG (Fig. 8a). Notably, the other 108 targets presented in metabolites more than 11 additional targets in SDG, indicating the potential pharmacological changes. Meanwhile, the protein–protein interaction network was also constructed (Fig. 8b and c). The main targets from PPI network were screened by analyzing their degree value in network, and proteins, with degree more than 3, were collected. Among them, PRKCB were the main targets of SDG (Fig. 8b), and the metabolites of SDG mainly targeted HSP90A1, IL6, AKT1, MAPK3, MTOR, PIK3CA, SRC, ESR1, AR, PIK3CB and PIK3CB (Fig. 8c). The difference of targets between SDG and its two metabolites could resulted in its functional changes of mechanism pathways or indications.

#### 3.4.1 KEGG classification of target proteins

The relationship between target proteins and the pathways was analyzed by using the data extracted from DAVID database, and the top 10 pathways were screened according to the KEGG analysis with BH-corrected *P*-values less than 0.05. The results showed that the main target proteins of SDG were mainly involved in inflammatory mediator regulation of TRP channels, vascular smooth muscle contraction, Fc gamma R-mediated phagocytosis, aldosterone synthesis and secretion, melanogenesis, and so on (Fig. 8d). Differently, the metabolites' target involved in neurotrophin signaling pathway, PI3K-Akt signaling pathway, pathways in cancer, prostate cancer and HIF-1 signaling pathway (Fig. 8e). This indicated that SDG might be used to treat prostate cancer or achieve its function through other signal pathways since the functional changes caused by its metabolites.

## 4. Conclusion

In this work, the metabolism feature and functional changes of SDG were explored by an integrated strategy based on metabolites profiling and network pharmacology. As a result, a total of 28 metabolites were found in rats, including 14 in plasma, 22 in urine, 20 in feces, 7 in heart, 14 in liver, 8 in spleen, 10 in lung, 14 in kidney, 4 in brain. Among them, M8, M13 and M26 were the main metabolites of SDG in rats and 24 of metabolites were characterized for the first time. Meanwhile, metabolism feature of SDG in rats was summarized, including the phase I reactions of demethylation, dehydroxylation, deglycosylation, arabinosylation and glycosylation and phase II reactions of glucuronidation, sulfation. Notably, the arabinosylation and glycosylation were found in SDG for the first time. Meanwhile, the targets and KEGG pathways of SDG and its metabolites were predicted and additional functional pathways of neurotrophin signaling pathway, PI3K-Akt signaling pathway, HIF-1 signaling pathway or indications of anti-prostate cancer were found. This work provided meaningful information for further pharmacological investigation of SDG and give a new sight for exploring the mechanism and therapy indications of other drugs, such as TCM.

## Conflicts of interest

There are no conflicts to declare.

## Supplementary Material

RA-010-D0RA06382G-s001
