# Supplementary material for: A combination of metabolite profiling and network pharmacology to explore the potential pharmacological changes of secoisolariciresinol-diglycoside
Source: RSC Adv. 2020 Sep 21;10(57):34847–58. doi: 10.1039/d0ra06382g (PMC9056848; doi:10.1039/d0ra06382g)
Supplement: RA-010-D0RA06382G-s001 [file RA-010-D0RA06382G-s001.pdf]

Electronic Supplementary Material (ESI) for RSC Advances.
This journal is © The Royal Society of Chemistry 2020



# A combination of metabolites' profiling and network pharmacology to explore the potential pharmacological changes of secoisolariciresinol-diglycoside in rats

Fengxiang Zhang[a, #], Yulinlan Yuan[a,#], Ziting Li[b], ShuangShuang Cui[a], Chang Li [c,*], Ruiman Li [a,*]

[a] Department of gynaecology and obstetrics, The First Affiliated Hospital of Jinan University, Guangzhou 510632, China
[b] Institute of Traditional Chinese Medicine and Natural Products, College of Pharmacy, Jinan University, Guangzhou 510632, China
[c] Department of Medicinal Chemistry and Natural Medicine Chemistry, College of Pharmacy, Harbin Medical University, Harbin 150081, China

[*]Corresponding to: Prof. Rui-man Li, Department of gynaecology and obstetrics, the First Affiliated Hospital of Jinan university, Guangzhou 510632, China, Tel: +86 020-38688603, fax: +86 020-38688603 E-mail: hqyylrm@126.com; Dr. Chang Li, Department of Medicinal Chemistry and Natural Medicine Chemistry, College of Pharmacy, Harbin Medical University, Harbin 150081, China, Tel: +86 0451-86685745, fax: +86 0451-86685745 E-mail: lichang661@126.com.

[#] The author contributes equally to this work

**Table S1** The area of SDG and its metabolites in rat's biosamples

| Name | Plasma | Urine | Feces | Liver | Spleen | Lung | Kidney | Heart | Brain |
|---|---|---|---|---|---|---|---|---|---|
| **P1** | 216.74 | 1881.65 | 5447.43 | 366.49 | 38.89 | 131.2 | 609.99 | 57.95 | 20.22 |
| **M1** | 0 | 141.36 | 11.12 | 0 | 0 | 0 | 0 | 0 | 0 |
| **M2** | 11.98 | 23.72 | 0 | 151.05 | 0 | 0 | 8.29 | 0 | 0 |
| **M3** | 0 | 0 | 121.04 | 0 | 0 | 0 | 0 | 0 | 0 |
| **M4** | 0 | 0 | 112.05 | 0 | 0 | 0 | 0 | 0 | 0 |
| **M5** | 10.86 | 0 | 0 | 148.91 | 0 | 0 | 15.05 | 0 | 0 |
| **M6** | 0 | 67.42 | 54.31 | 0 | 0 | 0 | 0 | 0 | 0 |
| **M7** | 0 | 1041.02 | 229.14 | 0 | 0 | 0 | 0 | 0 | 0 |
| **M8** | 12823.44 | 31464.42 | 309.47 | 11991.84 | 676.01 | 3181 | 6220.73 | 1379.56 | 389.29 |
| **M9** | 62.04 | 0 | 0 | 856.22 | 0 | 0 | 593.51 | 0 | 0 |
| **M10** | 105.17 | 0 | 0 | 330.51 | 0 | 0 | 144.44 | 0 | 0 |
| **M11** | 0 | 161.02 | 897.46 | 0 | 0 | 0 | 0 | 0 | 0 |
| **M12** | 0 | 188.02 | 1189.04 | 0 | 0 | 0 | 0 | 0 | 0 |
| **M13** | 90.98 | 18659.56 | 16888.38 | 1130.45 | 31.59 | 76.49 | 783.75 | 29.48 | 13.86 |
| **M14** | 439.99 | 115.83 | 0 | 202.53 | 12.64 | 73.85 | 468.01 | 48.47 | 0 |
| **M15** | 0 | 31.77 | 587.99 | 0 | 0 | 0 | 0 | 0 | 0 |
| **M16** | 0 | 0 | 184.35 | 0 | 0 | 0 | 0 | 0 | 0 |
| **M17** | 0 | 132.08 | 2743.81 | 0 | 0 | 0 | 0 | 0 | 0 |
| **M18** | 38.18 | 37.88 | 0 | 24.61 | 0 | 8.65 | 31.78 | 0 | 0 |
| **M19** | 0 | 150.63 | 405.39 | 0 | 0 | 0 | 0 | 0 | 0 |
| **M20** | 0 | 155.39 | 126.46 | 0 | 0 | 0 | 0 | 0 | 0 |
| **M21** | 44.2 | 179.26 | 344.84 | 842.62 | 9.79 | 28.87 | 462.12 | 0 | 0 |
| **M22** | 781.91 | 850.71 | 0 | 55.13 | 14.22 | 133.3 | 1368.63 | 50.99 | 9.31 |
| **M23** | 0 | 83.06 | 0 | 0 | 0 | 0 | 0 | 0 | 0 |

| | | | | | | | | | |
|---|---|---|---|---|---|---|---|---|---|
| **M24** | 0 | 179.44 | 8823.41 | 0 | 0 | 0 | 0 | 0 | 0 |
| **M25** | 77.83 | 318.41 | 260.43 | 1079.14 | 0 | 47.71 | 694.92 | 0 | 0 |
| **M26** | 984.6 | 2884.76 | 17260.35 | 1454.67 | 319.91 | 294.4 | 389.64 | 210.04 | 24.05 |
| **M27** | 16.34 | 0 | 1486.65 | 55.25 | 12.58 | 11.18 | 37.31 | 0 | 0 |
| **M28** | 33.29 | 143.26 | 1110.68 | 23.99 | 25.15 | 29.33 | 170.61 | 19.64 | 0 |
